# Exit Mechanisms of the Intracellular Bacterium *Ehrlichia*

**DOI:** 10.1371/journal.pone.0015775

**Published:** 2010-12-20

**Authors:** Sunil Thomas, Vsevolod L. Popov, David H. Walker

**Affiliations:** Department of Pathology and Center for Biodefense and Emerging Infectious Diseases, University of Texas Medical Branch, Galveston, Texas, United States of America; Tulane University, United States of America

## Abstract

**Background:**

The obligately intracellular bacterium *Ehrlichia chaffeensis* that resides in mononuclear phagocytes is the causative agent of human monocytotropic ehrlichiosis. *Ehrlichia muris* and *Ixodes ovatus* Ehrlichia (IOE) are agents of mouse models of ehrlichiosis. The mechanism by which *Ehrlichia* are transported from an infected host cell to a non-infected cell has not been demonstrated.

**Methodology/Principal Findings:**

Using fluorescence microscopy and transmission and scanning electron microscopy, we demonstrated that *Ehrlichia* was transported through the filopodia of macrophages during early stages of infection. If host cells were not present in the vicinity of an *Ehrlichia-*infected cell, the leading edge of the filopodium formed a fan-shaped structure filled with the pathogen. Formation of filopodia in the host macrophages was inhibited by cytochalasin D and ehrlichial transport were prevented due to the absence of filopodia formation. At late stages of infection the host cell membrane was ruptured, and the bacteria were released.

**Conclusions/Significance:**

*Ehrlichia* are transported through the host cell filopodium during initial stages of infection, but are released by host cell membrane rupture during later stages of infection.

## Introduction

The obligately intracellular bacterium *Ehrlichia chaffeensis* that resides in mononuclear phagocytes is the etiologic agent of human monocytotropic ehrlichiosis (HME). HME is an emerging and often life-threatening tick-transmitted infectious disease in the United States [1]. *Ehrlichia* are round or ovoid gram negative bacteria, and form a characteristic vacuole-contained microcolony (morula) in macrophages [2]. HME was first reported in 1987 [3]. Since then, development of murine models of persistent and lethal ehrlichiosis has greatly facilitated understanding of the pathogenesis and mechanisms of host defenses against ehrlichial infections. In general microorganisms can disseminate after host cell lysis via necrotic or apoptotic cell death, or by spreading from cell-to-cell [4]. The mechanism by which *Ehrlichia* are released from host cells has not been demonstrated [5]–[8].

Recently, in a mouse model of monocytotropic ehrlichiosis, we demonstrated by eastern blotting that the heat shock protein 60 (Hsp60/GroEL) is highly post-translationally modified in *E. muris,* which is not virulent in immunocompetent mice compared to the highly virulent strain IOE (*Ixodes ovatus* Ehrlichia) [9]. Based on this observation we generated an anti-*Ehrlichia* specific Hsp60 antibody and used it to observe *E. chaffeensis*, *E. muris* or IOE in cell culture. In this study we demonstrated by microscopic techniques the modes by which *Ehrlichia* exited the host cells.

## Results

### *Ehrlichia* are associated with the filopodia of infected DH82 cells

**Figure 1:**
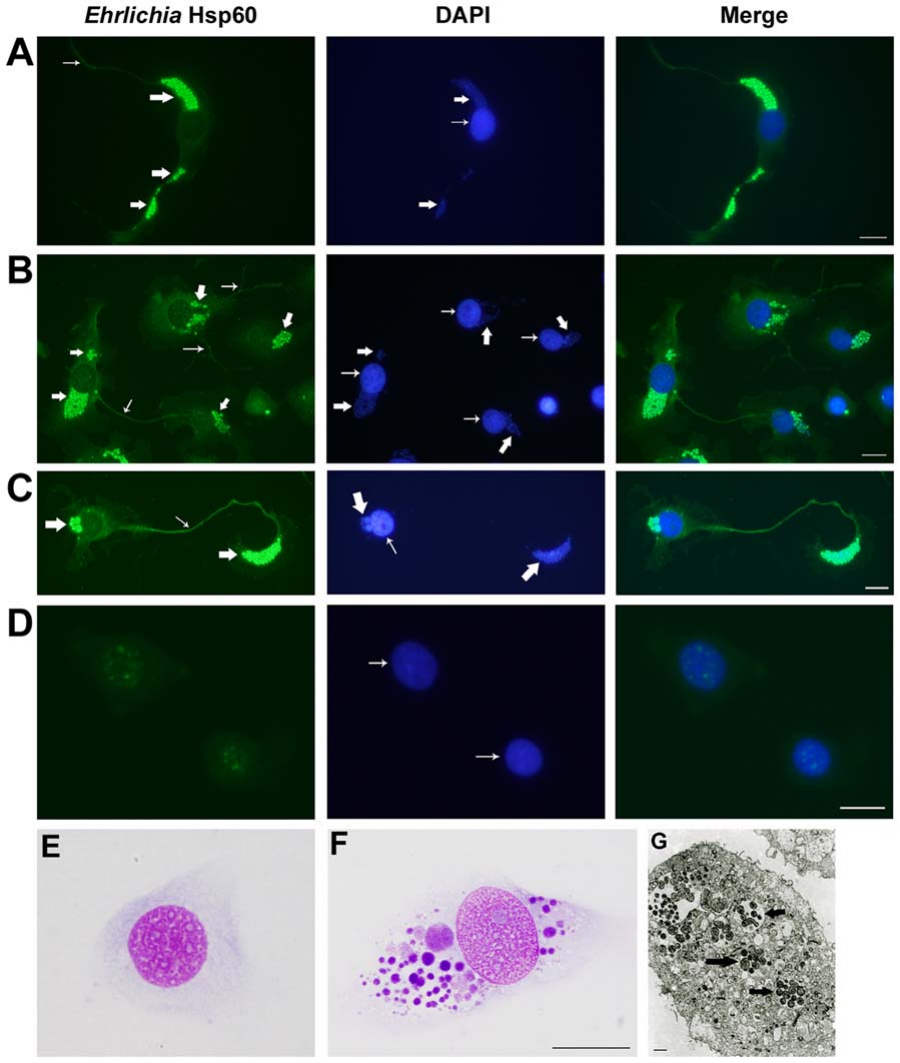
*Ehrlichia* are contained in the filopodia of DH82 cells. (A) Filopodia extended from the polar ends of the *E. chaffeensis-* infected DH82 cell. Left: *E. chaffeensis-*infected DH82 cell probed with anti-Hsp60 antibody. Thick arrow indicates *E. chaffeensis* intracellular colonies and thin arrow indicates filopodium. Middle: *E. chaffeensis-*infected cell stained with DAPI. Thick arrow indicates morulae of *E. chaffeensis* stained with DAPI and thin arrow indicates host nucleus. Right: Merged figure. Scale bar, 25 micrometers. (B) Filopodia of *E. chaffeensis-* infected DH82 cells extended to neighboring cells. (C) When host cells were not in the immediate vicinity, the leading edge of an *E. chaffeensis-* infected DH82 cell formed a flattened fan-shaped structure filled with the pathogen. (D) Uninfected DH82 cell. (E) Uninfected DH82 cell stained with Diff-Quik stain. (F) *E. muris-*infected DH82 cell stained with Diff-Quik stain. Scale bar, 25 micrometers. (G) Transmission electron micrograph of a DH82 cell infected with *E. muris*. Arrows indicate morulae of *E. muris*. Scale bar, 1 micrometer.

**Figure 2:**
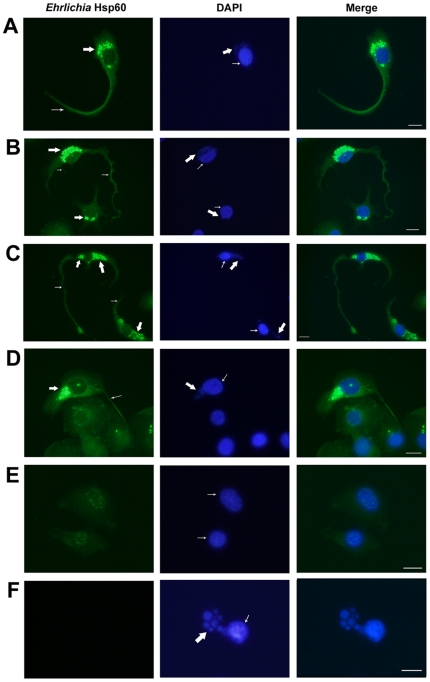
*E. muris* is associated with the filopodia of DH82 cells. (A–C) Filopodium extending from the cell body of an *E. muris-* infected DH82 cell. Left: *E. muris-*infected DH82 cell probed with anti-*Ehrlichia* Hsp60 antibody. Thick arrow indicates *E. muris,* and thin arrow indicates filopodium. Middle: *E. muris-*infected cell stained with DAPI. Thick arrow indicates DNA of *E. muris* stained with DAPI, and thin arrow indicates host nucleus. Right: Merged figure. Scale bar, 25 micrometers. (D) Filopodium of an *E. muris-* infected DH82 cell extended to a neighboring cell. (E) Uninfected DH82 cells. (F) Absence of *Ehrlichia* Hsp60 primary antibody resulted in absence of staining *E. muris* in infected DH82 cells, but DAPI stained the *E. muris* DNA and DH82 nucleus.

The intracellular pathogens *E. chaffeensis* and *E. muris* are maintained *in vitro* in the DH82 monocyte cell line. Previously *Ehrlichia* have been studied after infection of DH82 cells at high concentrations and culturing for 2–3 days when the host cells formed a confluent monolayer (no void between host cells). We observed *Ehrlichia* after seeding 1000–2000 infected DH82 cells per slide so that they were separated from one another (16 hours). *E. muris-* and *E. chaffeensis-*infected DH82 cells were probed with the anti-Hsp60 antibody. By 16 hours filopodia were observed in 30 percent of DH82 cells infected with *Ehrlichia* (3 percent in uninfected DH82 cells; p<0.0001). Filopodia extended from the polar ends of spindle-shaped *Ehrlichia-*infected host cells (*E. chaffeensis*: Fig. 1A, *E. muris*: Fig. 2A–D; uninfected DH82 cell: Fig. 1D, 2E; *E. muris-*infected DH82 cell without primary antibody: Fig. 2F) or from the non-polar sides of the cells when they contained many bacteria (*E. chaffeensis*: Fig. 1B). Filopodia of infected cells extended to the neighboring host cell (*E. chaffeensis*: Fig. 1B, *E. muris*: Fig. 2D). If host cells were not present in the vicinity of an infected cell, the leading edge of the filopodia of *Ehrlichia-*infected cells formed a flattened fan-shaped structure where the *Ehrlichia* morulae were contained (Fig. 1C). We have also observed that the morula-filled fan-shaped structure further developed its own filopodium (not shown). *Ehrlichia* infection in DH82 cells was confirmed using the Diff-Quik stain (Fig. 1F) and transmission electron microscopy (Fig. 1G).

**Figure 3:**
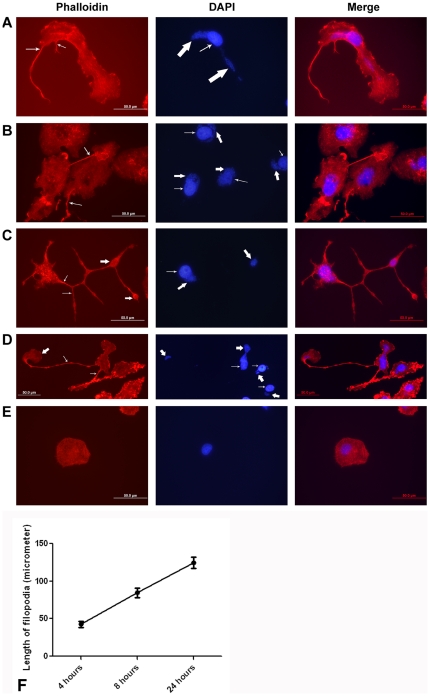
Actin was a major protein of filopodia induced during *Ehrlichia chaffeensis* infection. Left: *E. chaffeensis-*infected DH82 cell probed with phalloidin. Thin arrows indicate filopodia. Middle: *E. chaffeensis-*infected cell stained with DAPI. Thick arrow indicates morulae of *E. chaffeensis* stained with DAPI, and thin arrow indicates host nucleus. Right: Merged figure. Scale bar, 50 micrometers. (A) Filopodia extended from an *E. chaffeensis-*infected DH82 cell. (B) Filopodium of *E. chaffeensis-*infected DH82 cell extended to a neighboring cell. (C, D) *Ehrlichia* are contained in a long filopodium that had a flattened fan-shaped structure with no host cells in the immediate vicinity. Thick arrow indicates the flattened fan-shaped structure at the leading edge of the filopodium. (E) Uninfected DH82 cell. (F) Lengths of filopodia of DH82 cells infected with *E. chaffeensis* (n = 25).

Macrophage filopodia contain a meshwork of actin filaments and surround foreign organisms during phagocytosis [10]; cytochalasin D inhibits actin polymerization [11]. As phalloidin has a high affinity for actin, we used phalloidin conjugated to Alexa 594 for detection of actin in the filopodia. Filopodia stained with phalloidin-Alexa 594 were intensely red, whereas DAPI stained the host nucleus as well as the DNA of *E. chaffeensis* (Fig. 3A–D, uninfected DH82 cell: Fig. 3E). Filopodia formation was observed within an hour after culturing the infected DH82 cells. By 24 hours the average length of a filopodium in infected cells was 120 micrometers (Fig. 3F) (NS, p>0.05). We have observed filopodia in cell culture measuring more than 10 times longer than the diameter of the host cell.

**Figure 4:**
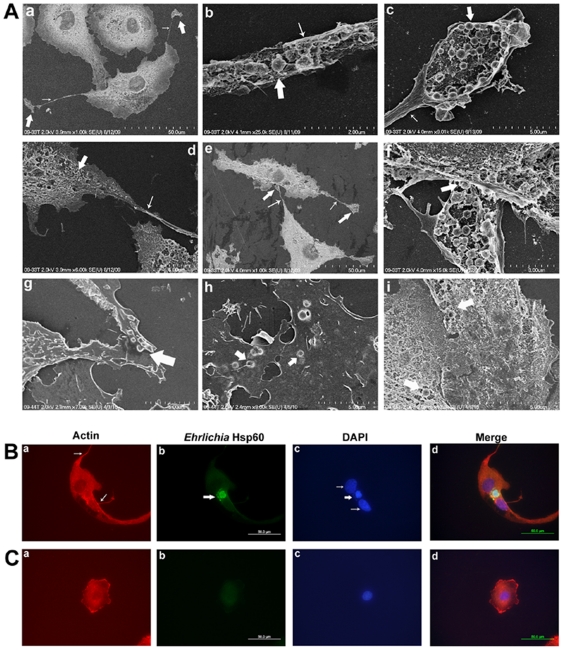
*Ehrlichia* is transported through the filopodia of the host cells. (A) Scanning electron micrographs of DH82 cells infected with *E. chaffeensis*. (a) *E. chaffeensis* are observed in the filopodia of DH82 cells. The thick arrow indicates the flattened fan-shaped structure at the leading edge of the filopodium (indicated by thin arrows). (b) *Ehrlichia* bacteria in a filopodium from which the cell membrane has been removed. The thick arrow indicates an *Ehrlichia*. (c) A flattened fan-shaped structure filled with *Ehrlichia* from which the cell membrane had been removed. The thick arrow indicates *Ehrlichia,* and the thin arrow indicates a filopodium. (d) A filopodium that extended from an *Ehrlichia-*infected DH82 cell. (e) Low magnification of an *Ehrlichia-*infected host cell filopodium in contact with a neighboring cell. The thick arrows indicate the flattened fan-shaped structures, and the thin arrows indicate the filopodia. (f) High magnification of a flattened fan-shaped structure from which the cell membrane has been removed at the leading edge of an *Ehrlichia-*infected cell (depicted in figure e) in contact with the neighboring host cell. The thick arrow indicates an *Ehrlichia.* (g) Intracellular *Ehrlichia* deforming the overlying cell membrane at the junction of a neighboring cell. (h) Localization of *Ehrlichia* (thick arrow) deforming the overlying cell membrane of adjacent cells. (i) *Ehrlichia* seen in adjacent cells of a cracked open DH82 host cell. (B) Actin was a major protein of filopodia during *E. muris* infection. (a) Thin arrows indicate filopodia; (b) thick arrow indicates *Ehrlichia* morula; (c) thick arrow in DAPI figure indicates *Ehrlichia* DNA, and the thin arrows indicate host nuclei and (d) merged figure. (C) Absence of filopodia in an uninfected DH82 cell.

Scanning electron microscopy of the mechanically opened cells demonstrated the presence of *Ehrlichia* in the filopodia of the DH82 host cells (Fig. 4Aa–f). On contact with a new cell, the pathogens from the fan-shaped flattened structure were in a location where they can pass to the neighboring cell (Fig. 4Ae–f). Further, observation of cell membranes deformed from within by intracellular ehrlichiae revealed the opportunity for bacterial intrusion into the adjacent cells (Fig. 4Ag–i). These observations suggest that *Ehrlichia* passed from one host cell to another without entering the extracellular space. To detect actin in the filopodium induced by *E. muris,* the infected DH82 cells were treated with anti-actin antibody. The concentration of actin was high in the filopodia of the infected DH82 cells (Fig. 4B), whereas a filopodium was rarely observed in the uninfected control cells (Fig. 4C).

### Inhibition of actin polymerization in DH82 cells infected with *Ehrlichia* prevented filopodia formation followed by the localization of the pathogen in the periphery of macrophages

**Figure 5:**
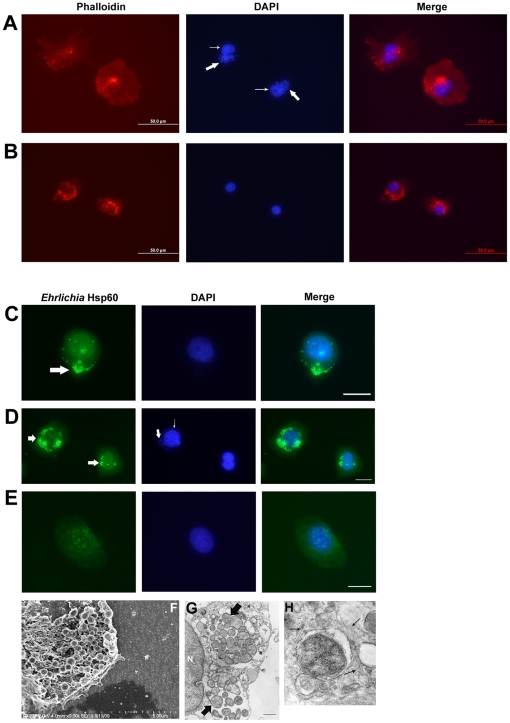
Cytochalasin D inhibited filopodium formation in *Ehrlichia-*infected cells. (A) *E. chaffeensis-*infected DH82 cells treated with cytochalasin D and stained with phalloidin (left), DAPI (middle) (thick arrows indicate *Ehrlichia* morulae and thin arrows indicate host nuclei), and merged figure (right). (B) Uninfected DH82 cells treated with cytochalasin D and stained with phalloidin. (C, D) *E. muris-* infected DH82 cells treated with cytochalasin D and probed with *Ehrlichia* Hsp60 antibody (left) (thick arrow indicates *Ehrlichia*), DAPI (middle) (thick arrow indicates *Ehrlichia* DNA, and thin arrow indicates host cell nuclei), and merged figure (right). (E) Uninfected DH82 cells treated with cytochalasin D and probed with *Ehrlichia* Hsp60 antibody. (F) Scanning electron micrograph of *E. muris-*infected DH82 cells treated with cytochalasin D from which the cell membrane had been removed. (G) Transmission electron micrograph of *E. muris-*infected DH82 cell treated with cytochalasin D. Thick arrows indicate *Ehrlichia* morulae, N, nucleus. Scale bar, 1 micrometer. (H) A single IOE cell in mouse spleen. Arrows indicate actin filaments.

Macrophages characteristically migrate and extend pseudopodia to assume an amoeboid conformation, whereas non-activated monocytes lack such processes and appear round [12]. Since actin and microtubules are involved in the formation of filopodia, we determined the effect of the actin inhibitor, cytochalasin D on the transport of the pathogen in *Ehrlichia-*infected monocytes. Cytochalasin D inhibited filopodium formation in both *E. chaffeensis-* (Fig. 5A) and *E. muris-* infected cells (Fig. 5C, D, F, G). Ehrlichiae were confined to the periphery of the macrophages. The experiments suggest the hypothesis that *Ehrlichia* are transported through the filopodia, a potential mechanism to avoid the host immune system while the pathogen passed from cell to cell.

**Figure 6:**
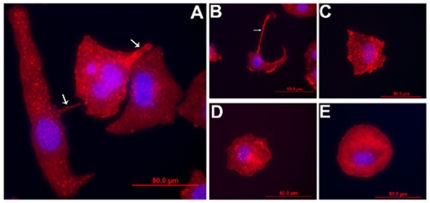
Actin inhibition prevented filopodium formation in *E. muris-*infected DH82 cells. (A) Untreated DH82 cells infected with *E. muris*. (B) Nocodazole treatment had no effect on filopodium formation. Treatment with (C) blebbistatin, (D) lantrunculin B, or (E) wiskostatin prevented filopodium formation.

Hybiske and Stephens [13] used latrunculin B (actin polymerization inhibitor), wiskostatin (N-WASP inhibitor) and blebbistatin (myosin II inhibitor) to demonstrate that actin, N-WASP and myosin are required for the non-lytic exit mechanism of the bacterium *Chlamydia*. Results from our studies also showed that latrunculin B, wiskostatin and blebbistatin inhibited filopodium formation; whereas treatment of *E. muris*-infected DH82 cells with nocodazole (microtubule inhibitor) did not inhibit filopodium formation (Fig. 6A–E).

**Figure 7:**
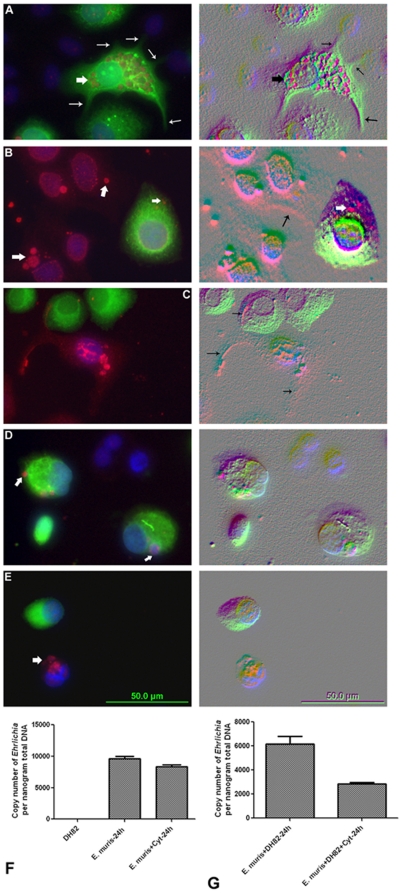
Inhibition of filopodium formation prevented *Ehrlichia* intercellular transport. (A) *Ehrlichia-*infected DH82 cells were treated with CFSE and seeded with uninfected non-treated DH82 cells for 24 hours. Thick arrow indicates *E. muris* (probed with *Ehrlichia* HSP60), whereas the thin arrows indicate filopodia/pseudopodia of infected cells. DAPI stains the nucleus of both the uninfected and infected cells. The adjacent figure is the Nomarski image, which clearly showed the filopodia/pseudopodia of infected cells. (B, C) DH82 cells were treated with CFSE and seeded with infected non-treated DH82 cells for 24 hours. Thick arrow indicates *E. muris* (probed with *Ehrlichia* HSP60) whereas the thin arrows indicate filopodia/pseudopodia of infected cells. DAPI stains the nuclei of both uninfected and infected cells. The adjacent figure is the Nomarski image which showed clearly the filopodia/pseudopodia of infected cells. (D) *Ehrlichia-*infected DH82 cells were treated with CFSE and seeded with uninfected non-treated DH82 cells for 24 hours in the presence of cytochalasin D. Thick arrow indicates *E. muris* (the adjacent figure is the Nomarski image). (E) Uninfected DH82 cells were treated with CFSE and seeded with infected non-treated DH82 cells for 24 hours in the presence of cytochalasin D. Thick arrow indicates *E. muris* (the adjacent figure is the Nomarski image). (F). Quantitative real time-PCR of bacterial loads of *E. muris-*infected DH82 cells to evaluate cytotoxicity in the presence of cytochalasin D (n = 3 per group). (G). Quantitative real time-PCR of bacterial load of *E. muris-* infected DH82 cells seeded with uninfected DH82 cells in the presence and absence of cytochalasin D (n = 3 per group).

Based on the findings that *Ehrlichia* are contained in the filopodium of DH82 cells, we hypothesized that filopodium formation is essential for intercellular transport of *Ehrlichia.* To test this hypothesis, we labeled *E. muris-*infected DH82 cells with carboxyfluorescein succinimidyl ester (CFSE) and seeded the infected cell culture with non-labeled uninfected DH82 cells. Alternately, we also labeled uninfected DH82 cells with CFSE and seeded with DH82 cells infected with *E. muris.* We observed filopodia/pseudopodia from the infected DH82 cells in close proximity to the neighboring uninfected DH82 cells and also observed *Ehrlichia* in the uninfected DH82 cells (Fig. 7A–C). In the presence of cytochalasin D we did not observe filopodia in the *Ehrlichia-*infected DH82 cells, and there was no infection in the neighboring uninfected cells (Fig. 7D–E). If *Ehrlichia* indeed are transported through the filopodia, we reasoned that the bacterial load will decrease in the presence of cytochalasin D when both infected and uninfected DH82 cells are seeded together as the pathogen cannot be transported to the uninfected cell. To test the hypothesis, we seeded uninfected DH82 cells with *E. muris-*infected DH82 cells (in the presence and absence of cytochalasin D) and analyzed the bacterial load by quantitative real time–polymerase chain reaction (RT-PCR) after 24 hours. Initial studies demonstrated that cytochalasin D was not toxic to *Ehrlichia* (Fig. 7F). When the uninfected DH82 cells were seeded with *E. muris-*infected DH82 cells in the presence of cytochalasin D, the bacterial load decreased, whereas in the absence of cytochalasin D the bacterial load increased (Fig. 7G).

### *Ehrlichia* are contained within the filopodia of infected mouse macrophages

**Figure 8:**
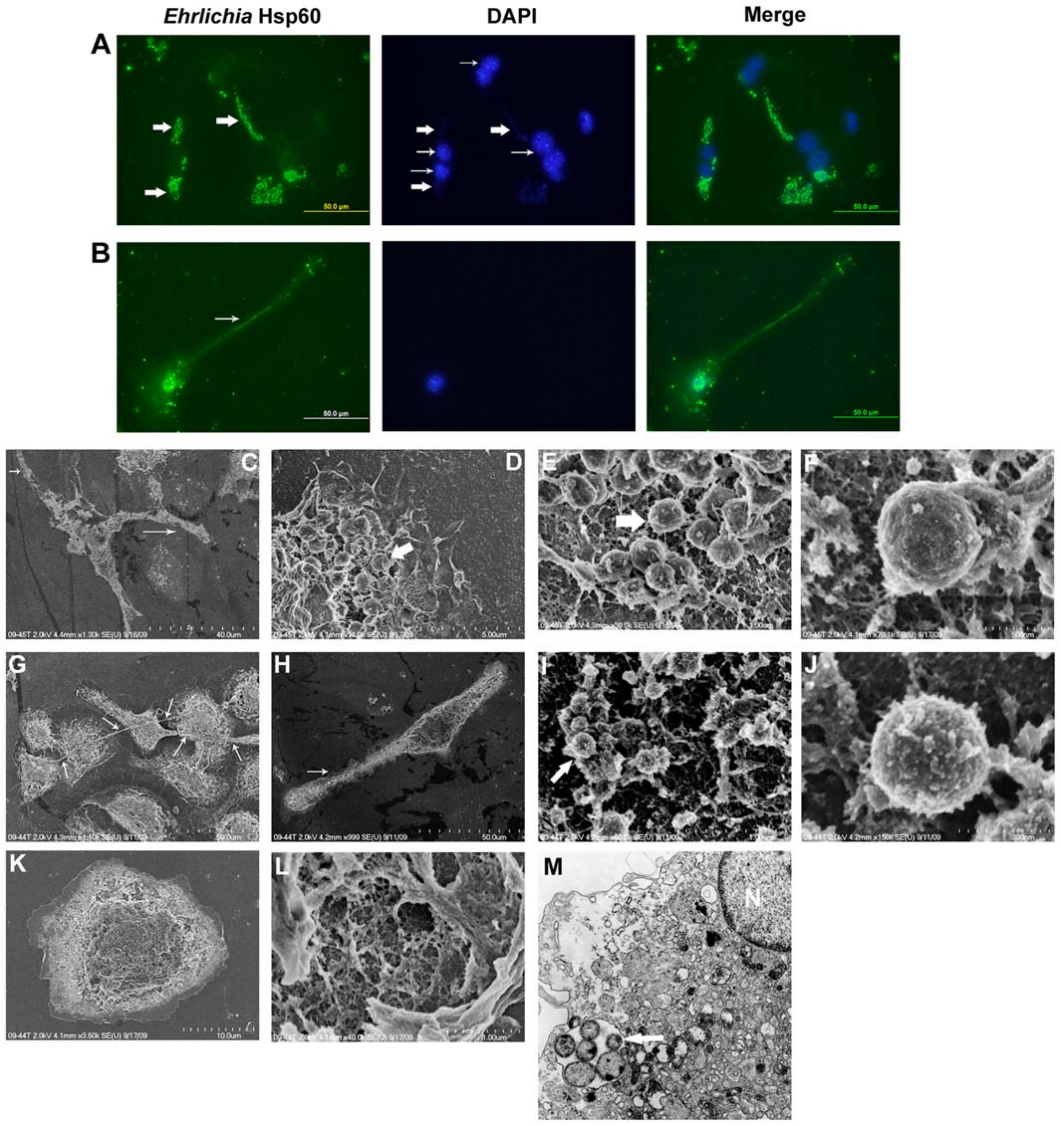
*Ehrlichia* are observed in the filopodia of mouse macrophages. (A) *E. muris-*infected mouse macrophages probed with *Ehrlichia* Hsp60 antibody (left), DAPI (middle) (thick arrows indicate DNA of *E. muris,* and thin arrows indicate mouse macrophage nuclei), and merged figure (right). (B) IOE-infected mouse macrophage probed with *Ehrlichia* Hsp60 antibody (left) (thin arrow indicates filopodium), DAPI (middle), and merged figure (right). (C) Scanning electron micrograph of *E. muris-*induced filopodium in a mouse macrophage; thin arrow indicates the filopodium. (D) The interior of a mouse macrophage from which the cell membrane has been removed contained *E. muris*. (E) Higher magnification of *E. muris* in a mouse macrophage. (F) Scanning electron micrograph of an *E. muris* bacterium. (G, H) Scanning electron micrograph of IOE-induced filopodia in mouse macrophages; thin arrows indicate the filopodia. (I) IOE microorganisms in a mouse macrophage. (J) Scanning electron micrograph of a single IOE bacterium. (K) Uninfected mouse macrophage. (L) High magnification of an opened uninfected mouse macrophage. (M) Transmission electron micrograph of a mouse macrophage that contained an *E. muris* morula (thick arrow), N, nucleus. Scale bar, 1 micrometer.

Lacking a technique to demonstrate the mechanism of *Ehrlichia* transport between cells *in vivo*, we cultured *in vitro* for five days splenocytes of mice that had been infected for 7 days prior to harvesting the cells. The cytoplasm of the macrophages from *E. muris-*infected mice harbored few pathogens on days 1–3 in cell culture, whereas by day 5 the macrophages were highly populated with *E. muris* (Fig. 8A, C–F, and M). *E. muris-*infected mouse macrophages had filopodia that contained the pathogen similar to those observed in the infected DH82 cells. Similar results were observed when macrophages from the highly lethal *Ixodes ovatus* Ehrlichia (IOE)-infected mice (infected for 7 days prior to harvesting) were cultured for five days *in vitro*. IOE were also observed in the filopodia of infected mouse macrophages (Fig. 8B, G and H).

### *Ehrlichia* infection led to a ruptured overlying host cell membrane at a late stage of infection

**Figure 9:**
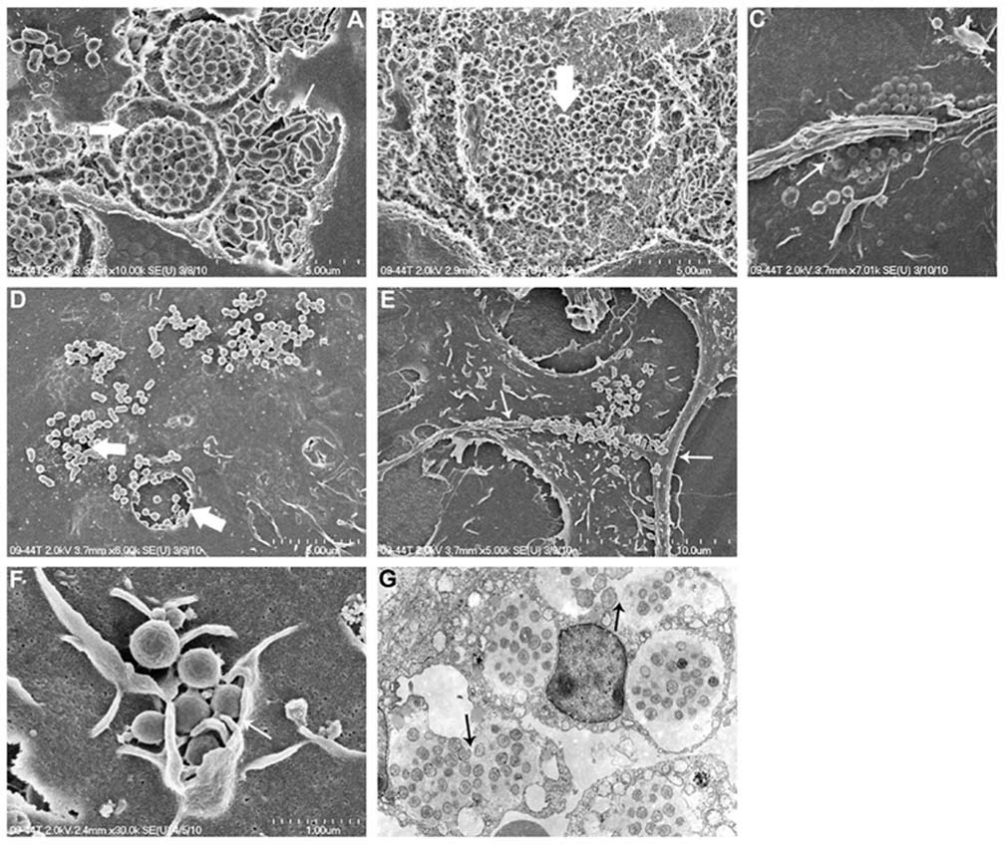
*Ehrlichia* morulae inside a DH82 cell with an overlying ruptured host cell membrane. (A) Different stages of *E. muris* in a mechanically opened DH82 cell. Thin arrow indicates dividing ehrlichiae; thick arrow indicates mature cells. (B) Mature ehrlichiae cells in a large morula (thick arrow). (C) Pore formation on a DH82 host cell containing many ehrlichiae that have deformed the overlying cell membrane (thin arrows) (intact DH82 cell). (D) Host cell membrane ruptured at the location of ehrlichial exit from the cell (intact DH82 cell). (E) Extracellular *Ehrlichia* attached with high affinity to the filopodium of neighboring host cells (thin arrows). (F) Ehrlichiae attached to the DH82 cell membrane adjacent to a cell membrane ruffle (thin arrow) (intact DH82 cell). (G) TEM of an IOE infected spleen (arrows indicate fused morula).

We cultured *E. muris* in DH82 cells for 60 hours on coverslips and observed specimens under a scanning electron microscope. At 60 hours the size of the morula enlarged probably due to fusion of adjacent morulae (Fig. 9B; Morulae of *E. muris* at 24 hours: Fig. 9A). The cell membrane of *E. muris* infected DH82 cell ruptured at 60 hours (Fig. 9C) and the bacteria were released through the pores on the host cell membrane (Fig. 9D). The pathogens released after membrane rupture were observed attached to the filopodium of neighboring cells (Fig. 9E). Attached ehrlichiae were observed in association with ruffled cell membrane characteristic of entry by endocytosis (Fig. 9F). TEM of IOE infected spleen confirmed morula fusion (Fig. 9G).

## Discussion

Successful establishment of infection by obligately intracellular bacterial pathogens requires adhesion to host cells, and cellular invasion followed by intracellular multiplication, dissemination to other tissues, or persistence [14]. An understanding of the specific pathways of pathogen exit is of fundamental importance to microbial pathogenesis because of its intimate association with dissemination, transmission and inflammation. Microbial exit is an organized and directed process mediated by both bacterial and cellular factors. *Shigella* and *Listeria* promote their escape from phagosomes through the action of pore-forming cytolysins: IpaB for *Shigella*, listeriolysin O and C-type phospholipases for *Listeria*. Cellular release then occurs as these bacteria use actin polymerization to protrude out of the cell [13]. Hybiske and Stephens [13] demonstrated that the intracellular bacteria, *Chlamydia,* are released by two mutually exclusive pathways-lysis and extrusion. The pathogenic *Mycobacteria* are also released by multiple exit mechanisms. Lasunskaia *et al*. [15] showed that *Mycobacteria* induce filopodia formation in macrophages. Recent studies by Hagedorn *et al.* [4] showed that *Mycobacteria* are released from the host cell by an actin-based ejectosome. The ejectosome was released into the phagocytic cup of the neighboring host cell.

The mechanism by which *Ehrlichia* exits the cell or is transported between cells is not known [7], [8]. In general, microorganisms are released by a lytic or non-lytic mechanism to infect neighboring host cells. This investigation demonstrated mechanisms by which *Ehrlichia* exited host cells. Our results based on microscopic analyses showed that *Ehrlichia* were contained in the filopodium of macrophages which were transported to neighboring cells. An advantage of *Ehrlichia* transport through the filopodia (in the initial stages) is that the pathogen evades the host immune system while it passed from cell to cell.

Filopodia are thin cell surface protrusions containing bundles of parallel actin filaments. Filopodia are designed for exploring the extracellular matrix and surfaces of other cells, identifying targets of adhesion, or navigating to its appropriate target. In epithelial cells they act as a zipper to fuse to one another [16]. Filopodia of the host cells have been shown to be utilized for cellular entry and exit by pathogenic microorganisms [17]. Bacteria also induce filopodia-like structures to enhance the cohesion of the bacterial microcolonies and therefore blood vessel infection under the harsh conditions of the bloodstream [18]. *Listeria* grow in the cytoplasm of macrophages and recruit and polymerize host cell actin, which provides the driving force for movement through the cytoplasm and into nearby cells by means of filopodia-like projections [19]. We have not observed ejectosomes or phagocytic cups as observed in *Mycobacteria-*infected host cells or a pinching off mechanism as observed in *Chlamydia-*infected host cells. We observed by electron microscopy *Ehrlichia-*containing cells with an extension to the neighboring cell mediated by the filopodia. Adhesion of one host cell to another mediated through the filopodium may be a mechanism of transport of *Ehrlichia*. Future investigations will focus on how ehrlichial proteins influence the host cell actin to induce filopodium elongation.

Once the host cell contains a large number of the bacterial pathogen, the host cell may not have a functional system which can induce actin polymerization for filopodium formation. Hence the only mechanism for the bacteria to exit the host cell is by lysing the host cell membrane. Blouin and Kocan [20] demonstrated the exit mechanism in *Anaplasma*. The authors showed that *Anaplasma* are released by membrane rupture of the host cell without apparent loss of host cell cytoplasm. Perforin-like proteins induce pore formation of host cells and are expressed by many bacterial and protozoan pathogens. Kafsack *et al*. [21] had demonstrated that *Toxoplasma gondii* secretes a perforin-like protein which disrupts the host cell membrane facilitating its exit. Perforin-like proteins are not documented in *Ehrlichia*; future studies may reveal if a similar protein is associated with the pathogen. Finlay and Falkow [22] had demonstrated that the actin cytoskeleton but not the microtubule network plays an active role in bacterial entry into host cells. SEM results (Fig. 9E) demonstrated that *Ehrlichia* have high affinity to the actin cytoskeleton. Based on our observation that phalloidin binds to the actin-rich filopodium, we hypothesize that the actin cytoskeleton may play a major role in *Ehrlichia* entry into host cells. Future study will determine the ehrlichial protein which binds actin cytoskeleton.

## Materials and Methods

### Cell culture

Three monocytotropic ehrlichial strains were used in this study. The human pathogen, *Ehrlichia chaffeensis* (a gift from Dr. Jere McBride, UTMB, Texas), and mildly virulent *E. muris* (provided by Dr. Y. Rikihisa, Ohio State University, Columbus, OH) were cultivated in DH82 cells [9] at 37°C in DMEM supplemented with 5% heat-inactivated bovine calf serum. Ehrlichiae were harvested when approximately 90 to 100% of the cells were observed to be infected. Infected cells were scraped, and 1000–2000 infected cells were cultured for 16 hours on culture slides. The non-culturable IOE was maintained in mice. The animals were sacrificed after 7 days and the spleen used as source of the pathogen.

### Fluorescence microscopy

Hsp60 (GroEL) peptides were synthesized by Biosynthesis, Inc. (Lewisville, Texas) and injected (i.p. - four times) into C57BL/6 mice. Antibody was obtained 40 days after the first injection. After fixation in 50% methanol-acetone, both *E. muris-* and *E. chaffeensis-*infected DH82 cells were incubated with the anti- *Ehrlichia* Hsp60 antibody (1∶125) (45 min), and after further washes they were reacted with anti-mouse immunoglobulin G conjugated to Alexa 488. After several washes they were mounted in mounting medium containing DAPI (Vectashield, Vector Labs, Burlingame, CA).

Phalloidin has high affinity for actin. Alexa 594-conjugated phalloidin (Invitrogen, CA) was incubated with *E. chaffeensis-*infected or uninfected DH82 cells following the manufacturer's instructions. Alternatively, for detection of actin, rabbit anti-actin antibody (1∶100) (Sigma, St. Louis, MO) was used for staining *E. muris*-infected or uninfected cells followed by treatment with goat anti-rabbit TRITC (1∶400). After several washes specimens were mounted in medium containing DAPI (Vectashield, Vector Labs, Burlingame, CA).

For filopodium inhibition studies, 30 minutes after transfer of *Ehrlichia*-infected DH82 cells to culture slides they were treated with cytochalasin D (0.5 micrograms/ml) (Fisher, Fairlawn, NJ). Cells were washed after 16 hours and fixed and stained as described above. Experiments were repeated three times. The cells were viewed by epifluorescence microscopy (Olympus BX51, Japan). For inhibition studies using the inhibitors lantrunculinB, wiskostatin, nocodazole, and blebbistatin, the method of Hybiske and Stephens [13] was followed. To determine that filopodia are essential for transport of *Ehrlichia* we treated infected or uninfected DH82 cells with CFSE (Invitrogen, CA) for 15 minutes following the manufacturer's instructions and seeded in the presence or absence of cytochalasin D. The cells were incubated for 24 hours and stained with *Ehrlichia* HSP60 antibody, further stained with anti-mouse Alexa 594, and mounted in medium containing DAPI (Vectashield, Vector Labs, Burlingame, CA).

### Determination of ehrlichial copy numbers in infected cells

Ehrlichial copy numbers in infected DH82 cells seeded with uninfected DH82 cells (in the presence or absence of cytochalsin D for 24 hours) were determined by quantitative real-time PCR method by analyzing the dsb gene [23].

### Electron microscopy

For transmission electron microscopy (TEM), the monolayers (*Ehrlichia-*infected cells) in T25 flasks were fixed in 2.5% paraformaldehyde and 0.1% glutaraldehyde in 0.05 M cacodylate buffer, pH 7.3, to which 0.03% trinitrophenol and 0.03% CaCl_2_ were added. After fixation the cells were washed with 0.1 M cacodylate buffer, scraped off the flasks and pelleted. The pellets were post-fixed in 1% OsO_4_ in 0.1 M cacodylate buffer, *en bloc* stained with 1% uranyl acetate in 0.1 M maleate buffer, dehydrated in ethanol and embedded in Poly/Bed 812 (Polysciences, Warrington, PA). Ultrathin sections were cut on Reichert-Leica Ultracut S ultramicrotome, stained with lead citrate and examined in a Philips 201 or CM-100 electron microscope at 60 kV.

For scanning electron microscopy (SEM), *Ehrlichia*-infected DH82 cells were cultured on Thermanox coverslips (Nunc, Rochester, NY) and were fixed similarly as for TEM, dehydrated in ethanol and processed through hexamethyldisilazane followed by air drying and mounting on stubs. The cells were mechanically opened with a touch of scotch tape (3M, St. Paul, MN) to expose the interior of the cells. Specimens were sputter-coated with iridium and observed with a Hitachi S4700 (Japan) scanning electron microscope.

### *In vitro* studies of mouse macrophages

*E. muris* (provided by Dr Y. Rikihisa, Ohio State University, Columbus, OH) or IOE (a gift from Dr M. Kawahara, Nagoya City Public Health Research Institute, Nagoya, Japan) were inoculated (i.p) into five week old C57/BL6 mice, and the animals were sacrificed after 7 days. The spleen was harvested, and the splenocytes cultured on a slide or flask in DMEM medium for 5 days. The infected or uninfected mouse cells were processed for epifluorescence microscopy, TEM and SEM. The mice were housed and cared for in the Animal Research Center at the University of Texas Medical Branch in accordance with the Institutional Animal Care and Use Committee guidelines under whose review and approval the experiments were conducted.

### Statistics

The experiments were performed in triplicate, and each figure is the representative of approximately 15–20 images.
